# The clinical and virological features of the first imported case causing MERS-CoV outbreak in South Korea, 2015

**DOI:** 10.1186/s12879-017-2576-5

**Published:** 2017-07-14

**Authors:** Ji Yeon Lee, You-Jin Kim, Eun Hee Chung, Dae-Won Kim, Ina Jeong, Yeonjae Kim, Mi-ran Yun, Sung Soon Kim, Gayeon Kim, Joon-Sung Joh

**Affiliations:** 1 Division of Pulmonary and Critical Care Medicine, Department of Internal Medicine, National Medical Center, Seoul, 04564 Republic of Korea; 2 Korea Centers for Disease Control and Prevention, Cheongju, 28159 Republic of Korea; 3 Division of Pediatric Allergy & Pulmonology, Department of Pediatrics, Chungnam National University School of Medicine, Chungnam National University Hospital, Daejeon, 35015 Republic of Korea; 4 Center for Infectious Diseases, National Medical Center, Seoul, 04564 Republic of Korea

**Keywords:** Middle East respiratory syndrome Coronavirus, Genetic analysis, Complete genome, Index case, Korea

## Abstract

**Background:**

In 2015, the largest outbreak of Middle East respiratory syndrome coronavirus (MERS-CoV) infection outside the Middle East occurred in South Korea. We summarized the epidemiological, clinical, and laboratory findings of the first Korean case of MERS-CoV and analyzed whole-genome sequences of MERS-CoV derived from the patient.

**Case presentation:**

A 68-year-old man developed fever and myalgia 7 days after returning to Korea, following a 10-day trip to the Middle East. Before diagnosis, he visited 4 hospitals, potentially resulting in secondary transmission to 28 patients. On admission to the National Medical Center (day 9, post-onset of clinical illness), he presented with drowsiness, hypoxia, and multiple patchy infiltrations on the chest radiograph. He was intubated (day 12) because of progressive acute respiratory distress syndrome (ARDS) and INF-α2a and ribavirin treatment was commenced. The treatment course was prolonged by superimposed ventilator associated pneumonia. MERS-CoV PCR results converted to negative from day 47 and the patient was discharged (day 137), following rehabilitation therapy. The complete genome sequence obtained from a sputum sample (taken on day 11) showed the highest sequence similarity (99.59%) with the virus from an outbreak in Riyadh, Saudi Arabia, in February 2015.

**Conclusions:**

The first case of MERS-CoV infection had high transmissibility and was associated with a severe clinical course. The patient made a successful recovery after early treatment with antiviral agents and adequate supportive care. This first case in South Korea became a super-spreader because of improper infection control measures, rather than variations of the virus.

## Background

The largest outbreak of Middle East respiratory syndrome coronavirus (MERS-CoV) outside the Middle East occurred in South Korea in May 2015: the first confirmed case of a patient who recently visited endemic areas. Over the following 2 months, 186 cases were confirmed, including 36 deaths [1].

Of 24 imported cases of MERS in countries outside the Middle East, only 4 cases involved at least one secondary transmission event and 4 transmitted cases, excluding South Korea [2, 3]. It is important to evaluate the cause of this significant outbreak in South Korea, and whether the clinical features of the first patient were different from those in patients in other countries.

The National Medical Center (NMC), Seoul, Korea, was designated as the primary MERS response center (MERS Countermeasure Headquarters) and treated patients with MERS, including the first patient. We report the clinical course and laboratory findings of the first case of MERS-CoV in South Korea to understand the unexpected spread of the outbreak. Unlike previous papers focused on epidemiology [4], we focused on detailed clinical progress and genetic analysis that have not been previously published.

## Case presentation

### Sources of data

History of potential exposure to MERS-CoV was elicited by direct interview with the patient and his/her family members. Clinical and laboratory findings were obtained from the medical records.

### Genome sequencing

RNA samples extracted from a sputum sample from the index patient on day 11 (May 22, 2015) were reverse transcribed to cDNA followed by PCR with MERS-CoV-specific primer pairs with overlaps. Amplicons were pooled for library preparation, and next generation sequencing was performed using the MiSeq sequencing system. The genomic sequences of the virus were assembled using CLC genomic workbench 8.0.1, and the obtained 29,995-bp whole genome sequence was registered in GenBank as MERS-CoV/KOR/KNIH/001_05_2015 (accession no. KT326819).

### Phylogenetic construction and ORF comparison

All previously published 93 MERS-CoV complete genome sequences and the Korean index case were aligned using MUSCLE v3.8.31. Phylogenetic analyses were performed using RAxML v8.1.21 (1000 bootstrap replicates), with the GTR model of nucleotide substitution and γ-distributed rates among sites. From the phylogenetic tree, 8 representative sequences, including each lineage and year, were selected for further analysis. The amino acid sequence identity of 10 open reading frames (ORF) between index sequence and 8 reference sequences of MERS-CoV was determined using BLAST. Each 10 ORF sequence was aligned using the MUSCLE program and all amino acid changes for each ORF sequences were analyzed. A root-to-tip regression analysis was performed with Path-O-Gen software to investigate the temporal signal in the data (http://tree.bio.ed.ac.uk/software/pathogen/). Phylogeny without a molecular clock was inferred from the maximum likelihood method implemented in RAxML, and then analyzed in Path-O-Gen with the dated tips.

### Clinical history

**Fig. 1:**
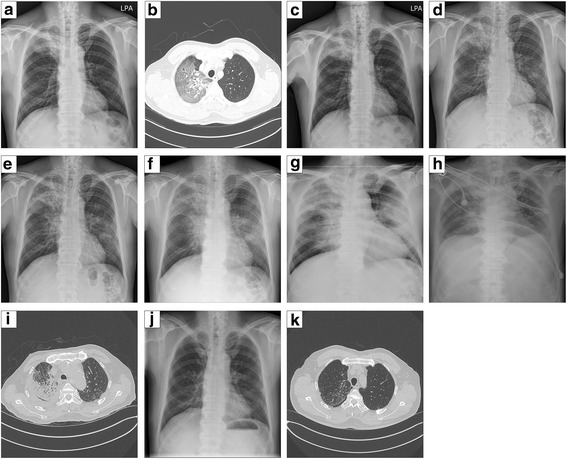
Imaging changes in the first case of Korean MERS-CoV infection outbreak. Panel **a** and **b** (May 15, 2015) shows diffuse ground-glass opacity infiltration and consolidation in central portion of right upper lung lobe. Panel **c**-**g** (May 16–20, 2015) shows aggravation of the multiple patchy pulmonary infiltration in right lung and left middle lung field. Panel **h** and **i** (July 3, 2015) show peribronchial infiltration and collapse consolidation of the right upper lobe and pleural effusion in right. Panel **j** and **k** (June 15, 2016) show that infiltrations in both lungs are generally improved compared to previous findings but fibrosis and distorted bronchial thickening remain in *right upper lobe*

A 68-year-old Korean man developed fever and myalgia on May 11, 2015 (D0) in Korea. He visited a local clinic (A) in Pyeongtaek, Gyeonggi province on D1, 3 and 4. Laboratory results on D3 were within normal ranges, except for mild lymphopenia (Table 1). He was prescribed ribostamycin, acetaminophen, afloqualone, rebamipide, and streptokinase; however, the fever and myalgia persisted. On May 15 (D4), he developed a nonproductive cough. He was hospitalized in a private general hospital (B) in Pyeongtaek, Gyeonggi province between 15 and 17 May. Chest radiographs and computed tomography of the chest (D4) showed a diffuse ground-glass opacity and consolidation in the central portion of the right upper lobe of the lung (Fig. 1a and b).

**Table 1:** Laboratory data for the first case of Korean Middle East Respiratory Syndrome coronavirus outbreak

	Normal range	D3	D4	D5	D7	D8	D9	D11	D12	D13	D14	D15	D17	D19
Hemoglobin (g/dL)	13.0 ~ 17.0	13.9	13.5	12.6	14.5	13.3	13.7	12.1	10.9	10.7	12.1	11.9	10.5	9.9
WBC (cells /uL)	4000 ~ 10,000	3900	2710	2500	3330	3220	3400	3100	4000	7500	8300	9100	6500	8100
Neutrophils (%)	50 ~ 75	69	68.4	56.4	73.3	75.5	80.4	77.8	79.5	89	87	88.6	77.2	77.8
Lymphocytes (%)	20 ~ 44	24	23.5	36.8	21.3	18.6	14.5	15.1	11.6	5	6	5.6	12.3	11.9
Platelet count (103 cells /uL)	130 ~ 400		111	98	139	140	160	168	142	114	56	81	122	162
BUN (mg/dL)	5 ~ 20	11.1	10.5		8.3	11.1	9	9	8	8	12	14	19	31
Creatinine (mg/dL)	0.5 ~ 1.2	1.1	0.94		0.98	0.94	1	0.8	0.7	0.9	0.7	0.7	0.7	0.8
AST (U/L)	0 ~ 40	41	44	56	164	141	176	146	87	54	34	31	56	93
ALT (U/L)	0 ~ 45	28	30	35	90	82	97	102	75	57	39	33	34	60
Albumin (g/dL)	3.3 ~ 5.2		3.4				2.5	2	1.7	1.7	1.5	1.4	1.9	2.1
Total bilirubin (mg/dL)	0.2 ~ 1.2		0.37		1.1	0.7	1.1	1.2	1.4	1.8	4.2	3.4	3.2	2.2
C-reactive protein (mg/dL)	0 ~ 3.0		7.26	7.26		7.92		9.1	8.23			13.9	9.54	

*Abbreviations*: *D* day, *WBC* White blood cell count, *BUN* blood urea nitrogen, *AST* aspartate aminotransferase, *ALT* alanine aminotransferase

Ceftriaxone and amikacin were administered intravenously for 3 days; however, symptoms did not improve. The patient was discharged on May 17 (D6) and attended the emergency department (ED) of a university hospital (D) in Seoul, via another clinic (C), in Pyeongtaek. Owing to overcrowding, he was discharged from the ED after radiography and blood tests were performed, and returned the following day. On D7, oxygen saturation (O_2_Sat) was 91% on room air, although the patient did not complain of dyspnea. He was placed on 1 L/min of oxygen (O_2_) via a nasal cannula and prescribed ceftriaxone and azithromycin for community-acquired pneumonia, as well as oseltamivir, for possible influenza, and admitted to the medical floor of the hospital (D). Chest radiographs (D5–9) showed increasing bilateral multifocal pneumonic consolidation (Fig. 1c-g). The multiplex PCR results of a nasopharyngeal swab taken on D7 were negative for adenovirus, influenza virus (A/B), parainfluenza virus (1/2/3), respiratory syncytial virus (A/B), human metapneumovirus, human rhinovirus (A/B/C), coronavirus (229E/OC43/NL63), and bocavirus. Microbiological tests were also negative for anti-*Mycoplasma pneumoniae* antibodies, and urine antigen tests were negative for pneumococcus and *Legionella pneumophila* (D7). Blood and sputum cultures were negative (D7). On D8, sputum samples were tested for MERS-CoV and a positive result was confirmed the following day. The patient was transferred to NMC on May 20 (D9).

### Medical history

The patient had 10-year and 5-year histories of hypertension and dyslipidemia, respectively. He had no history of diabetes mellitus, pulmonary tuberculosis, or allergies. His medications included irbesartan, atorvastatin, and aspirin. He was a 50 pack-year smoker, and drank alcohol rarely.

### Exposure history

The patient was a businessman living in South Korea and occasionally visiting the Middle East on business. On April 24, 2015, he flew from South Korea to Bahrain, via Doha, Qatar. He stayed in Bahrain until May 3, 2015, except for two single-day business trips to the United Arab Emirates and to Saudi Arabia. He runs a business in Bahrain, providing greenhouse systems for professional growers. During his stay he was the sole occupier of a second floor apartment in a five-story building, with two native households residing on each floor. The building was a 15-min drive from the worksite. There was no elevator in the building and he never met the neighbors. He went to work in his own car and did not use public transportation. In Bahrain, he supervised business in the field, attended meetings, and met many buyers from nearby countries. He did not encounter any ill persons and there were no camels or bats in proximity of the greenhouse.

On April 29, he visited Abu Dhabi for meetings with buyers. On May 1, he went to Saudi Arabia for a greenhouse construction site inspection. He moved to the outskirts of Riyadh by private car and was in contact with ~50 greenhouse workers for ~4 h. He never visited any location other than Riyadh while staying in Saudi Arabia.

On May 4, he returned to South Korea from Bahrain, via Doha.

During his stay in the Middle East, he did not have any contact with unwell persons and did not visit any hospitals. He also denied physical contact with or consumption of camels or camel products, directly or indirectly via other persons.

### Transmission history [4–8]

**Fig. 2:**
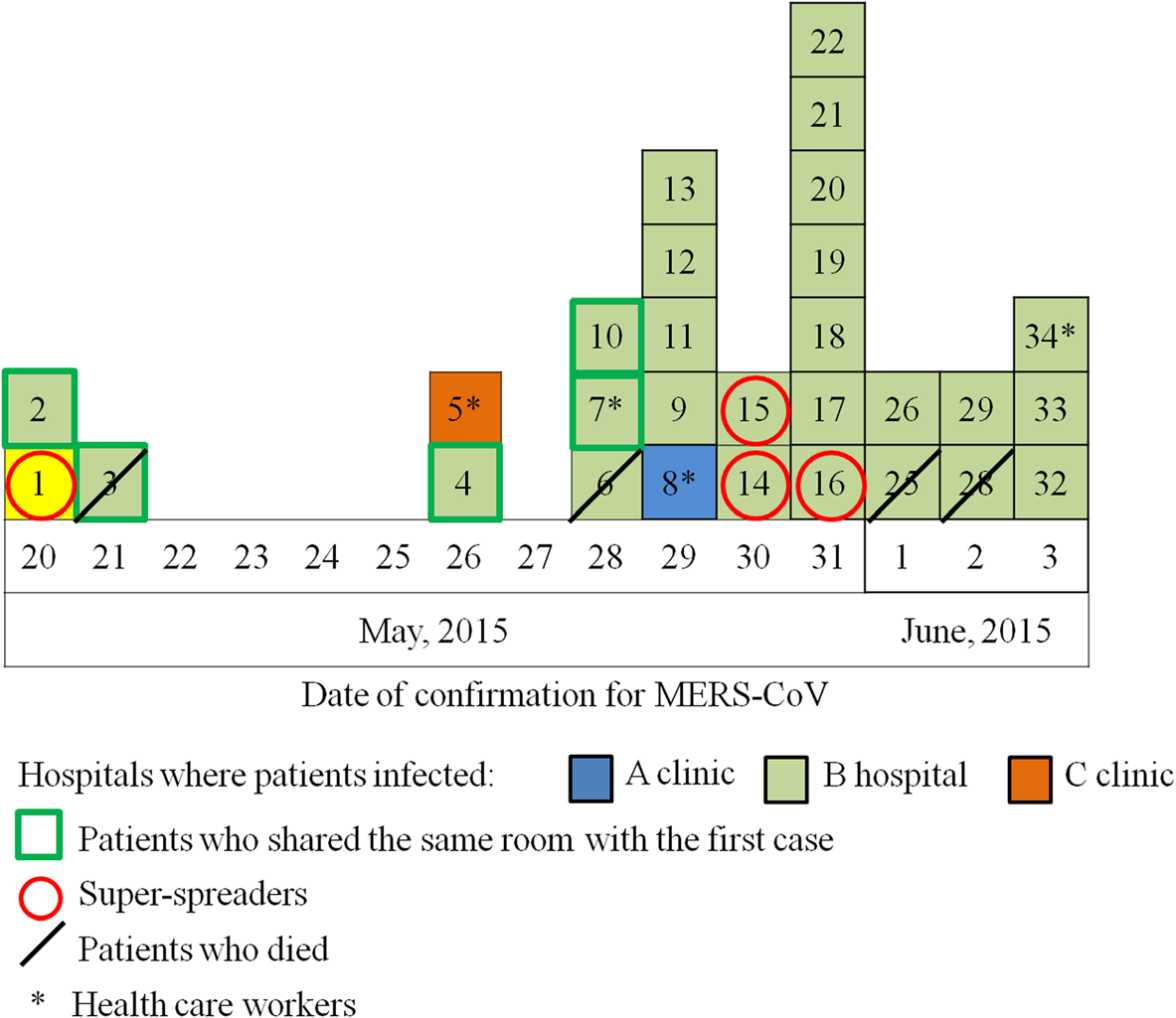
Transmission of MERS-CoV associated with the first case of the Korean MERS-CoV infection outbreak [4, 8, 32]

Twenty-eight secondary cases have been traced back to the primary case (Fig. 2), of which, 5 involved death due to MERS and 23 discharges from hospital following successful treatment. The first patient did not undergo nebulizer treatment. He circulated within the same ward and in the area surrounding the hospital. He did not visit other rooms/wards.

### Clinical course

On admission to NMC on May 20 (D9), the patient reported a fever, chills, generalized myalgia, malaise, cough, and dyspnea. His body mass index was 27.68 kg/m^2^. He appeared drowsy and confused and O_2_Sat was 94% on 2 L/min of O_2_ via nasal cannulae.

Laboratory data on admission revealed lymphopenia, elevated liver function tests, hypoalbuminemia, and elevated inflammatory markers (Table 1). Chest radiography showed multiple bilateral patchy pulmonary infiltration (Fig. 1g).

The patient was initially placed on 3 L/min of O_2_ via nasal cannulae; however, his O_2_ requirement increased to 5 L/min (D10). He was intubated (D12) for progressive hypoxia and ARDS. Bronchoscopy performed immediately after intubation showed no endobronchial lesion and a slightly brownish secretion from the anterior segmental bronchus of the right upper lobe.

**Fig. 3:**
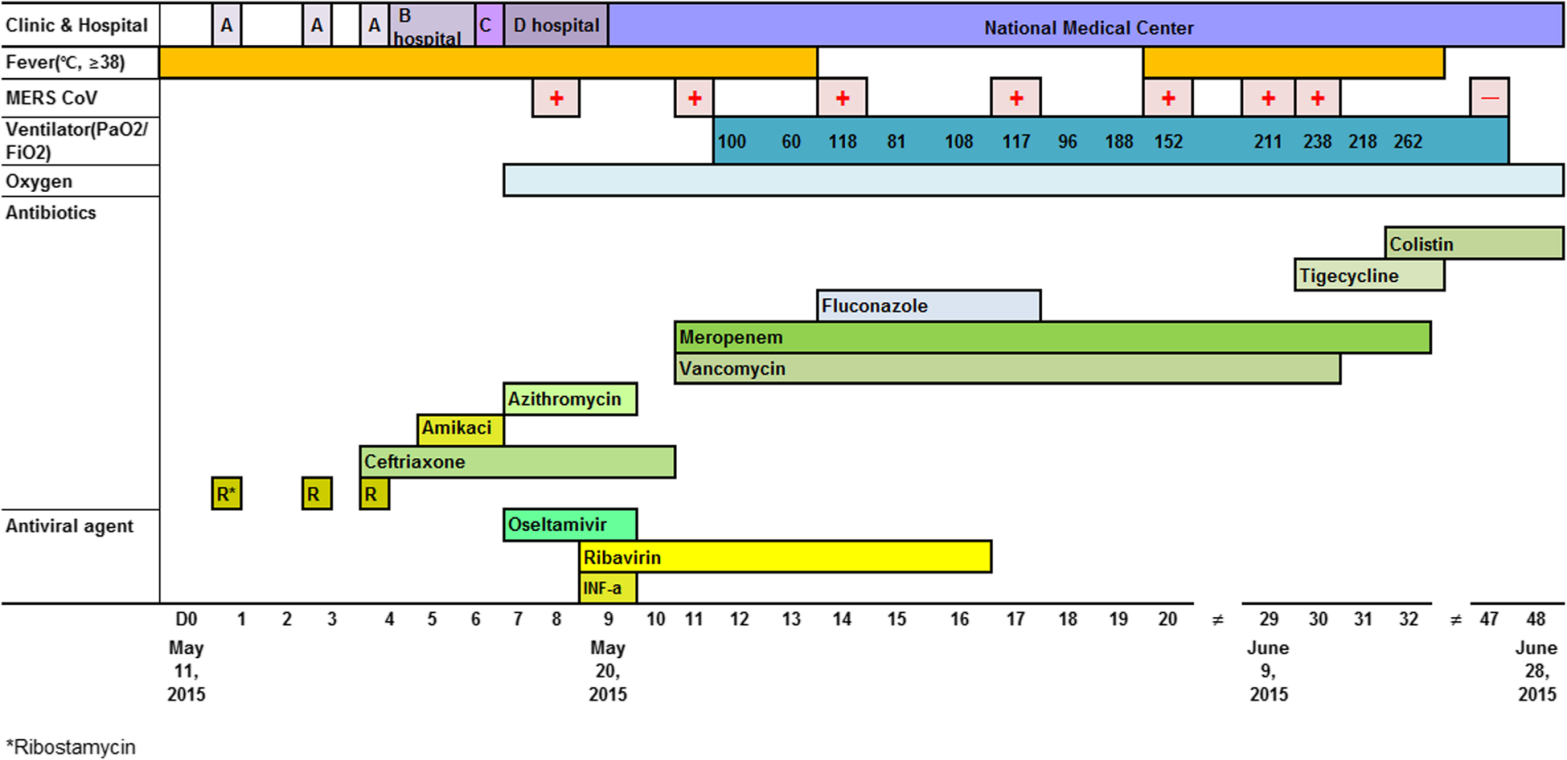
Hospital course and treatment of the first case of the Korean MERS-CoV outbreak

Pegylated interferon-α2b 180 mcg once (D9), and oral ribavirin (D9–16; 2000 mg loading dose, 600 mg q 8 h for 3 days, and 400 mg q 8 h for 4 days) were prescribed. Intravenous ceftriaxone and azithromycin were continued until D10 and escalated to vancomycin and meropenem on D11, because of clinical aggravation. Fever persisted for 14 days (D0–13), with a maximum temperature of 39.6 °C on D12, and then subsided from D14 (Fig. 3).

The patient developed a fever again (D20) and a maximum temperature of 39.5 °C persisted until D27. C-reactive protein levels decreased to 3.5 mg/dl on D24, but increased to 15.7 mg/dl on D28, with aggravation of leukocytosis and purulent secretions. Ventilator associated pneumonia was suspected. Tigecycline was added (D30) and meropenem was replaced by intravenous colistin (D32), as *Acinetobacter baumannii*, resistant to meropenem, was detected on endotracheal aspiration performed on D27, 33, 34, and 35. Bronchoscopy (D30) showed a large amount of purulent secretions occluding the right middle and lower lobar bronchi. Bronchoscopic toileting was performed daily (D30–39). On D32, tracheostomy was performed, and the patient was weaned off the ventilator on D37. The fever subsided on D38. MERS-CoV PCR results were inconsistently positive until D44 and then converted to negative for over three consecutive days (D47, 50, 51). On D53, the patient no longer required O_2_ (O_2_Sat of 98–100% on room air). He was discharged on September 25 (D137) after sacral sore treatment and rehabilitation therapy. Nine months later, follow-up pulmonary function tests and a chest CT scan (Fig. 1k) showed marked improvement: FVC 4.89 L (104% predicted); FEV 1 3.84 L (119% predicted); FEV 1/FVC 79%; and diffusing capacity of the lung for carbon monoxide (DLCO) 14.2 mL/min/mmHg (65% predicted).

### Infection control procedures

The patient wore a surgical mask during transfers. Medical staff wearing personal protective equipment accompanied him to NMC. He entered hospital through an emergency door with a separate hallway, using an emergency elevator. He was admitted to an isolated ward into a negative-pressure room with airborne and contact precautions. He received all treatment, including ventilator care, bronchoscopic treatment, and tracheostomy, in the same negative-pressure room, before being released from quarantine.

### MERS-CoV genome analysis

The genome of the index case (KT326819) consisted of 29,995 nucleotides with a ploy(A) tail and 10 ORFs were predicted. The nucleotide sequence identity by pairwise comparisons using the 93 complete genome sequences from the NCBI and the public site was in the range of 97.75–99.59%. The MERS-CoV of the index case produced the best similarity (99.59%) with the virus from an outbreak in Riyadh, Saudi Arabia, in February 2015 (Riyadh_KKUH_0780_20150225) included in lineage 5. In amino acid sequences comparisons, Riyadh_KKUH_0780_ 20150225 also show high identities through 10 ORFs (99.1–100.0%) (Table 2). They encoded five identical proteins (ORF3, E, M, N and ORF8b). When compared with 8 representative strains, most ORFs showed high identities (over 99.0%) and the E gene was identical to each strain. ORF3 was the most variable gene with identities of 96.1–100.0%. The S gene encoding the spike protein which interacts with the host receptor was identical, ranging from 99.8 to 99.9%.

**Table 2:** Percentage identity between ORFs of the Korean strain and human MERS-CoV representative strains at the amino acid level

Lineage	Strain	ORF1ab	S	ORF3	ORF4a	ORF4b	ORF5	E	M	N	ORF8b
Outgroup	EMC/2012	99.6	99.9	96.1	99.1	99.6	100.0	100.0	99.1	99.8	99.1
Lineage 1	England-Qatar/2012	99.8	99.8	98.1	100.0	100.0	100.0	100.0	99.1	99.3	100.0
	MERS − CoV − Jeddah − human − 1	99.7	99.9	95.2	99.1	99.6	100.0	100.0	99.5	100.0	99.1
Lineage 2	Al-Hasa_1_2013	99.8	99.9	97.1	99.1	100.0	100.0	100.0	99.1	100.0	100.0
	Abu-Dhabi_UAE_8_2014	99.7	99.8	98.1	99.1	100.0	98.7	100.0	99.1	99.8	-
Lineage 3	Riyadh_2014KSA_683/KSA/2014	99.6	99.9	98.1	100.0	99.2	100.0	100.0	99.5	99.5	100.0
Lineage 4	Jeddah_C7770/KSA/2014–04-07	99.8	99.9	98.1	100.0	100.0	100.0	100.0	99.1	99.5	97.3
Lineage 5	Riyadh_KKUH_0780_20150225	99.9	99.9	100.0	99.1	99.2	99.6	100.0	100.0	100.0	100.0

**Fig. 4:**
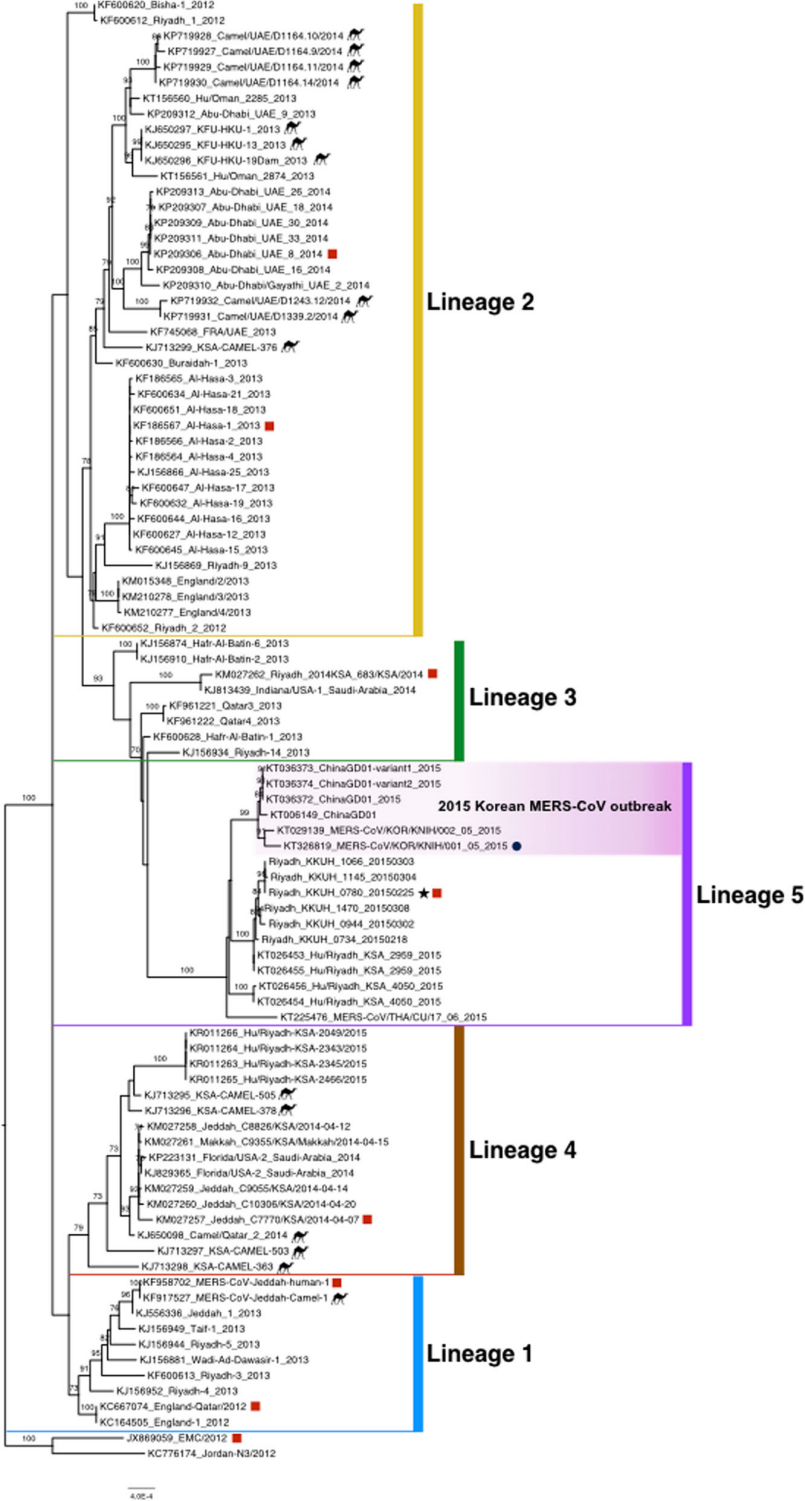
The maximum likelihood tree was estimated in RAxML from the 94 MERS-CoV genome dataset. Values on branches are bootstrap values from the nucleotide. Only bootstrap values ≥70% are shown as percentages on the basis of 1000 replicates. The *black* dot indicates the index case. The star spot indicates the closest strain among complete genome sequences. Branch lengths are proportional to the number of substitutions per site (see *scale bar*). The *highlighted red* squares indicate the representative sequence used in the further sequence analysis. The camel MERS-CoV sequences are labeled with a camel icon

In phylogenetic analysis, the Korean strain was clustered in the lineage 5 of locally spreading 2015 Riyadh strains, but distinguished from Riyadh with a bootstrap value of 99 (Fig. 4). This result could be owing to the fact that the 2015 Riyadh strains, including Riyadh_KKUH_0780_20150225, were the closest relatives and may be the origin of the Korean strains.

**Fig. 5:**
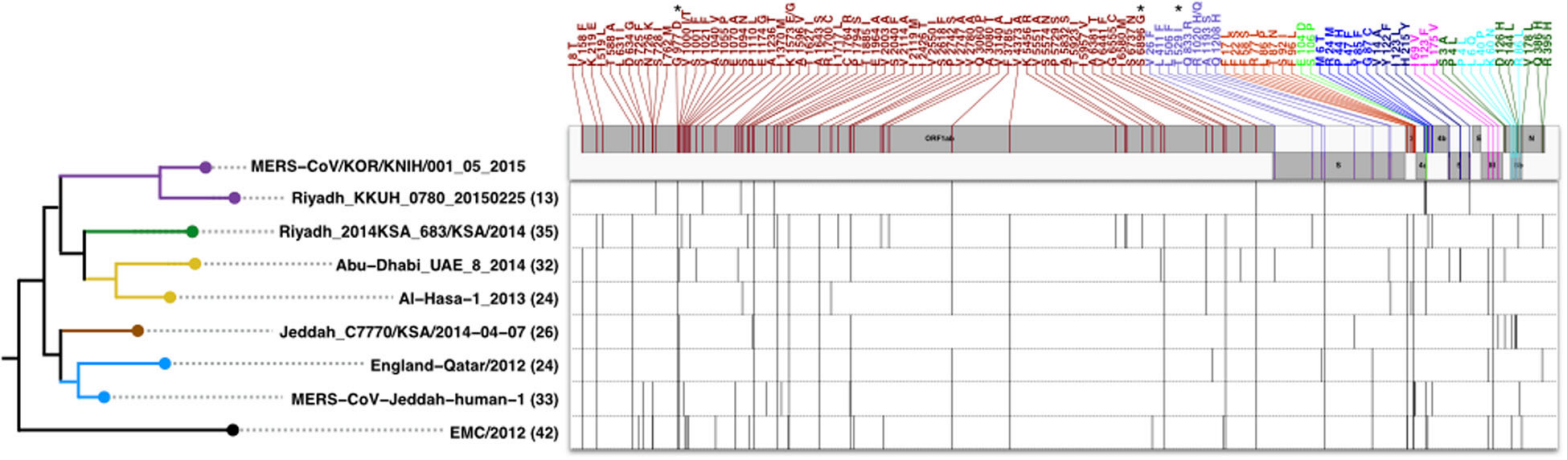
Phylogenetic tree and sequence alterations. The *left* panel shows the phylogenetic relation of eight sequences selected from each lineage which were constructed using the maximum likelihood method in MEGA6 with the following settings: GTR + I. The branches with different *colors* show their lineages depicted in Fig. 4. Each strain name is linked to the branch with a *dotted line*. The number in parentheses is the alteration count in each strain compared to the Korea index strain. The *right panel* shows non-synonymous (NS) single-nucleotide differences (*vertical colored bars*) between the Korean index genome and the representative genomes available. Mutation information is displayed as follows: change in ORF1ab (*red*), change in S protein (*orange*), change in ORF3 (*yellow*), change in ORF4a (*green*), change in ORF4b (*blue*), change in ORF5 (*navy*), change in M (*purple*), change in N (*dark green*) and change in ORF8b (*sky*). The *asterisk* indicates unique alterations in the Korean index strain

To identify the genetic features separated from the 2015 Riyadh strains, we analyzed amino acid changes between the Korean index genome and 8 isolates in representative lineage. We found 104 amino acid alteration sites. Compared to the EMC/2012 strain, the Korean index case showed 42 amino acid differences, whereas the closest Riyadh_KKUH_0780_20150225 strain showed 13 amino acid inconsistencies (Fig. 5). When we examined the 93 reference whole genomes, we found previously unreported amino acid changing alteration sites, in the ORF1ab (D977G, G6896S) and the receptor-binding domain (RBD) of S gene (I529T). In addition to these nonsynonymous alterations, 8 unique synonymous alteration sites were identified in the ORF1ab (T7154, C9956T, C10994T, T11927C, C18790A), S (T24632C, C24722T) and ORF3 (C25714T) gene.

**Fig. 6:**
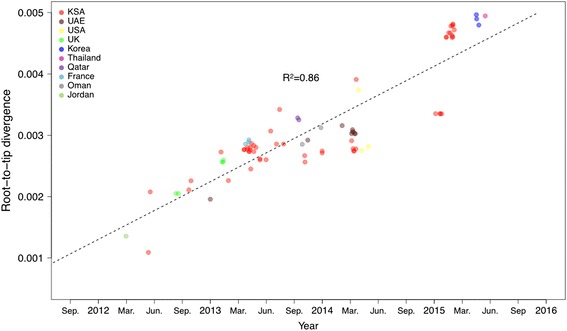
Root-to-tip genetic divergence plotted against the sampling date. The genetic distances from the root were calculated on the maximum likelihood phylogeny. Each y-axis value represents the genetic distance from the tip to the root, and the x-axis value represents the corresponding sampling date of the tip. The points and regression *dotted line* shown were obtained from the ML tree presented in Fig. 4. The R^2^ value is indicated above the *regression line*

We showed that genomic change occurs in the MERS-CoV Korean strains. We further examined whether these evolution rates exhibit spatial and temporal patterns during outbreaks. We plotted root-to-tip distances of the isolates against geographic location and isolation time (Fig. 6). The root-to-tip method measures rates over the entire history of a lineage, and is suitable for comparing evolution rates to long-term sequence traits. We observed a strong and statistically significant phylogeographic correlation between mutation rate and isolation date, recognizing the tendency that the root-to-tip distances through time were increased. For example, the isolates in the 2013–2014 year of MERS-CoV displaying shorter root-to-tip distances were in closer proximity to EMC/2012 than the 2015 Korean and Thailand isolates. The regression line showing this tendency was marked as a dotted line and the previously reported sequences were closely fitted to line (R2 = 0.86). The y values of the 2015 Korean strains and closest Riyadh strains of KSA were slightly higher than the regression line, meaning that these strains had changed faster than the average strains. However, the distances from the regression line were on the range of other past MERS-CoV strains. This suggested that the rate of evolution or adaptation of the Korean strains to the host were not the considerably different and MERS-CoV had undergone gradual change. The Korean MERS-CoVs showed some genetic differences compared to previous strains. To explain the meaning of each nucleotide change and the influence on viral transmissibility or disease severity, further studies are required.

## Discussion

Like previous MERS outbreaks, the MERS-CoV infection in Korea was hospital acquired transmission. However, the overwhelming extent arouse suspicion that there might be a distant feature at the molecular, genetical, clinical, and epidemiological level. It is postulated that the first case in South Korea transmitted MERS-CoV to 28 secondary cases [4–8]. There have been no reports of such a significant outbreak in countries outside the Middle East experiencing importation of MERS-CoV [3]. In the 2013 outbreak in Saudi Arabia, one patient transmitted MERS-CoV to seven other persons, raising concerns about a potential super-spreader [9]. In the Korean outbreak, 5 patients, including the first case, were reported to be super-spreaders, transmitting the virus to 153 out of 186 MERS patients [1]. What makes certain hosts become super-spreaders remains unclear. Host, pathogen, and environmental factors may be implicated in the MERS outbreak [10]. In the first case, prolonged duration of exposure before diagnosis and proper isolation and frequent inter-hospital transfer may have contributed to the super-spreading event. Higher pathogen shedding may also have contributed to the spread because the patient developed severe pneumonia and the severe cough and high viral load could facilitate infection transmission. Additionally, nosocomial transmission by healthcare workers or environmental factors, such as inadequate ventilation, could be responsible. Kim, et al. reported that the MERS-CoV was confirmed by RT-PCR of viral cultures of air samples and environmental surface swabs from hospital B, suggesting the possibility of contact and airborne transmission [11]. In line with previous results, MERS-CoV contamination of environmental surfaces [12] and virus persistence in the air surrounding the infected camels or humans, persisted for at least 24 h [12, 13].

The possibility of increased strain virulence was raised in the Korean outbreak; however, there was no evidence of viral mutation capable of modifying the mode of transmission or virulence until now [14–17]. Firstly, the identified Korean strains showed no specific evidence of viral adaptation to change infectivity or transmissibility. The 2015 Riyadh strains, which are a recombinant from the EMC_2012 strain and Jeddah-1_2013, were suggested as the ancestor of Korean strains [18]. Several variation sites, particularly in spike proteins, have been identified but these variations including I529T, firstly found in Korean index patient, showed that the affinity for the host receptor was reduced, resulting in reduced entry efficiency [16]. Furthermore, during infection in patients or in transmission chains, no evidence of adaptive changes and of rapid changes in the evolutionary rate was found [15].

Here, the complete genome of the index case was newly reported and analyzed. The index patient stated that he had not contacted symptomatic persons, camels, or bats in the course of his business trip to Riyadh, on May 1. However, a phylogenetic tree strongly suggested that the MERS-CoV was transmitted from the circulating Riyadh strains.

Many patients were infected with MERS-CoV in hospitals A, B, and C, although no confirmed secondary cases of infection occurred in hospital D while the index patient remained in ED for 2 days, for 4 and 5 h, respectively, before placement into isolation [19]. This difference may be explained by various factors, such as patient behavior and symptoms or degree of contact exposure. Many secondary cases occurred in hospital D, at a later stage; however, these resulted from transmission by another patient [19].

The patient manifested the most prevalent MERS symptoms: fever (98%), chills (87%), and cough (72–83%) but no gastrointestinal tract symptoms, generally observed in over 20% of MERS patients. Laboratory data were comparable to those of other MERS patients, including lymphopenia, elevated liver enzymes, and inflammatory markers. Transient progression of thrombocytopenia from D12 appeared to be caused by interferon and ribavirin [20, 21]. Pneumonia progressed rapidly and ventilator support was required. Studies have shown that the clinical features of MERS are similar to those of SARS; however, MERS progresses to respiratory failure much more rapidly. Specifically, the fatality rate of MERS is much higher and is likely to be associated with older age and comorbidities [22].

In the absence of approved specific therapy, clinical management of MERS largely depends on supportive care and prevention of complications. Our patient also received supportive care, such as O_2_ therapy, mechanical ventilation, inotropic support, and antimicrobial therapy. Although there is no proven effective antiviral therapy against MERS-CoV, in vitro data suggest that combined use of interferon and ribavirin reduces disease severity in a rhesus macaque model of MERS [23]. In a retrospective study of 20 patients with MERS, ribavirin and interferon-α2a improved 14-day and 28-day survival by 70 and 28%, respectively, compared to an untreated group [24]. However, another study found that there were no survivors among 5 patients with MERS receiving a combination therapy of interferon-α2b and ribavirin. The median time from admission to therapy for these patients was 19 (range, 10–22) days, suggesting that late administration of antiviral therapy may have contributed to the poor outcome [25]. Therefore, timing antiviral therapy may be critical. In our patient, antiviral therapy was initiated on admission.

Duration of treatment of the index case was more prolonged because of the development of secondary bacterial pneumonia. Multidrug-resistant pathogens, such as multidrug-resistant *A. baumannii*, were persistently isolated from D27. Previous studies have also reported nosocomial bacterial infections in patients with MERS receiving invasive mechanical ventilation, including infection with *Klebsiella pneumoniae*, *Staphylococcus aureus*, and *Acinetobacter* species [26–28]. High prevalence rates, reaching 65%, of nosocomial pneumonia during ARDS have been observed, [29, 30]. It is unclear whether patients with ARDS are more susceptible to pneumonia or have more risk factors [31]. Considering the infection risk, tracheostomy could not have been performed earlier in our case, resulting in prolonged mechanical ventilation.

Despite the severe clinical course, our patient made a full recovery. Follow-up pulmonary function tests normal, except for a slightly decreased DLCO. Studies examining lung function outcome and longer-term consequences and sequels of MERS survivors have not been reported until recently; therefore, further follow-up is needed.

## Conclusions

In summary, the first case of MERS-CoV infection had high transmissibility and a severe clinical course, although the patient made a successful recovery. Specialized intensive care may be critical in the treatment of MERS, because patients often progress to ARDS requiring ventilatory support. Unlike MERS cases imported from other countries, our first case became a super-spreader because of the social practices in Korea, rather than variations of the virus. The Korean health care system is vulnerable to hospital-acquired infection and proper infection control measures are needed to prevent MERS-CoV transmission in the healthcare setting.

## Abbreviations

ED: Emergency department
MERS-CoV: Middle East respiratory syndrome coronavirus
NMC: National Medical Center
O_2_: Oxygen
ORF: Open reading frames
